# A New Model for Screening for Late-Onset Preeclampsia in the Third Trimester

**DOI:** 10.3390/jcm14207185

**Published:** 2025-10-12

**Authors:** Clara Jiménez-García, Ana María Palacios-Marqués, José Antonio Quesada-Rico, Paloma Baviera-Royo, Encarnación Pérez-Pascual, Inmaculada Baldó-Estela, Víctor García-Sousa

**Affiliations:** 1 Department of Clinical Laboratory, Dr. Balmis General University Hospital, 03010 Alicante, Spain; 2 Department of Obstetrics and Gynecology, Dr. Balmis General University Hospital, 03010 Alicante, Spain; apalacios@umh.es (A.M.P.-M.); paloma.baviera@gmail.com (P.B.-R.); eperezpascual62@gmail.com (E.P.-P.); baldo.inm43@gmail.com (I.B.-E.); vicgarso@gmail.com (V.G.-S.); 3 Alicante Institute for Health and Biomedical Research (ISABIAL), 03010 Alicante, Spain; 4 Department of Public Health, History of Science and Gynecology, Miguel Hernández University, 03550 San Juan de Alicante, Spain; 5 Department of Clinical Medicine, Miguel Hernández University, 03550 San Juan de Alicante, Spain; jquesada@umh.es; 6 Network for Research on Chronicity, Primary Care and Health Promotion (RICAPPS), 03550 San Juan de Alicante, Spain

**Keywords:** late preeclampsia, third-trimester screening, predictive model, angiogenic factors

## Abstract

**Background/Objectives**: Screening for late-onset and term preeclampsia (PE) is essential, as the early identification of women at high risk enables closer monitoring and reduces adverse outcomes. The existing algorithms combining maternal factors, biophysical and biochemical markers have not been validated outside the populations in which they were originally developed. This study aimed to evaluate the predictive performance of the Fetal Medicine Foundation (FMF) third-trimester algorithm in our population and develop a novel model to improve the predictions. **Methods**: An observational, analytical, prospective cohort follow-up study was conducted at the Health Department of Alicante, Dr. Balmis General University Hospital, including 1580 singleton pregnancies recruited between February 2022 and November 2023 during routine third-trimester ultrasounds. Maternal clinical characteristics, blood pressure, the uterine artery pulsatility index (UtA-PI), and the sFlt-1/PlGF ratio were recorded. The FMF third-trimester algorithm was retrospectively applied at the end of pregnancy using clinical, biophysical, and biochemical data from 30 + 0 to 37 + 6 weeks via the freely accessible online calculator. The data analysis was performed using SPSS v.28 and R v.4.3.1. **Results**: A total of 1580 women were included, with a prevalence of late-onset PE of 2.9%. The FMF model achieved an area under the curve (AUC) of 0.87 (95% CI: 0.81–0.92), while our own model showed a superior performance, with an AUC of 0.94 (95% CI: 0.92–0.97). **Conclusions**: The FMF third-trimester algorithm demonstrated a good predictive performance for late-onset PE. Our newly developed model achieves an even higher predictive accuracy and offers a simplified approach to excluding the UtA-PI, which facilitates its use in routine clinical practice.

## 1. Introduction

Preeclampsia (PE) is a pregnancy-specific condition characterized by gestational hypertension associated with proteinuria or maternal organ dysfunction or uteroplacental dysfunction after 20 weeks of gestation. PE, as a significant cause of maternal and neonatal morbidity and mortality, is one of the most noteworthy obstetric complications [1,2]. In recent years, it has been shown that PE not only poses an immediate risk to the health of the mother and the fetus but also has significant medium- and long-term repercussions. Recent studies have demonstrated that women who have suffered from PE and their children are at greater risk of developing cardiovascular and renal diseases in the future [3,4].

Two types of PE are defined based on the time of onset: early, when delivery occurs before 34 weeks, and late, when delivery takes place at 34 weeks or later. The importance of this classification extends beyond labeling the disease since these subtypes differ in their pathophysiology, associated complications, and clinical management [5,6]. The incidence of PE increases with gestational age, meaning that late PE accounts for 70% of cases in developing countries and 90% of cases in developed countries [7].

Most efforts have focused on predicting early PE. However, screening for late and term PE is important since identifying pregnant women at high risk allows for closer monitoring and the most appropriate time and place for delivery to be determined, thereby minimizing adverse maternal and perinatal outcomes [8]. Furthermore, identifying women at high risk of late PE not only contributes to better prenatal care but also allows a group of women at higher risk of developing cardiovascular diseases to be recognized and the appropriate preventive measures to be initiated.

Screening for late and/or term PE can be performed using algorithms applied during the first, second, or third trimester of pregnancy. Several algorithms have been proposed for use in the first trimester, including those by Kuc et al. [9], Scazzocchio et al. [10], and Crovetto et al. [11]. Among these, the model developed by Scazzocchio et al. [10] has undergone external validation in four independent cohorts across different countries, including the United States, Spain, and the United Kingdom. Notably, one of these validations was a single-center study conducted at the Hospital Clínic of Barcelona. The remaining models have each been validated only once. Examples of predictive models for use in the second trimester include those developed by Zanello et al. [12] and Gallo et al. [13]. Among models designed for the third trimester, an important example is the algorithm proposed by Andrietti et al. [14], which corresponds to the FMF model. This model incorporates maternal factors, mean arterial pressure (MAP), uterine artery pulsatility index (UtA-PI), placental growth factor (PlGF), and soluble fms-like tyrosine kinase-1 (sFlt-1). It was externally validated in the STATIN study (Döbert et al. [15]). The results of studies validating these algorithms indicate that the detection rates increase when screening is conducted at later gestational stages [16,17]. Cohort studies have reported average detection rates of 39% and 50% in the first and second trimesters, respectively. Detection rates increase markedly in the third trimester, reaching 61% when screening is conducted early in the third trimester (30–34 weeks) and 76% in the late third trimester (35–37 weeks). These data indicate that performing screening at more advanced stages of pregnancy may significantly improve the predictive performance of models for identifying late-onset and/or term PE. Nevertheless, there is limited information available on the use of combined models in the second and third trimesters. Several groups have developed multivariable algorithms that combine clinical parameters, biochemical markers such as sFlt-1 and PlGF, and biophysical markers (such as blood pressure and the mean uterine artery pulsatility index) to facilitate the prediction of late PE [16]. However, most of these models have not been validated in populations of pregnant women other than those in which they were developed [18]. The prevalence of this condition, as well as the specific characteristics of pregnant women, differs according to geographic area, which could affect the external validity of these models in terms of disease detection rates. In Spain, the prevalence of late PE is estimated to be approximately 2.6% [11], a rate comparable to that observed in other European populations and developed countries [8]. In contrast, in low- and middle-income countries, the overall prevalence of PE is generally higher, reaching up to 4% [19]. Thus arose the need to carry out this study, in which the decision was made to validate the competing risk algorithm from the Fetal Medicine Foundation (FMF) for the third trimester of pregnancy [8] since it had not previously been validated in a Spanish population. This could represent a useful tool for late PE screening in our pregnant population. At the same time, the development of our own predictive model was proposed, with the aim of optimizing its predictive capacity in comparison with that of preexisting algorithms.

## 2. Materials and Methods

### 2.1. Study Design

Our observational, analytical, prospective cohort follow-up study was carried out among pregnant women at the Health Department of Alicante, Dr. Balmis General University Hospital, between February 2022 and November 2023.

### 2.2. Patients

A required sample size of 1581 pregnant women was estimated using the Epidat^®^ program version 4.2. Assuming an annual population of approximately 1800 pregnant women delivering at the Dr. Balmis General University Hospital and an expected prevalence of late-onset PE of 1.7% (based on the hospital’s 2019 data: overall PE prevalence = 2.19%, early-onset PE = 0.47%, late-onset PE = 1.72%), with a 95% confidence level, a precision of 0.3%, and an anticipated 10% loss to follow-up, the estimated sample size required was 1581 pregnant women.

The participants selected for this study met all of the following inclusion criteria: pregnant women aged ≥16 years; a singleton pregnancy with a live fetus; pregnancy monitored at the Obstetrics Service of the Health Department of Alicante, Dr. Balmis General University Hospital; and delivery at the same hospital.

The exclusion criteria were multiple pregnancies; pregnancies in which the fetus presented major morphological anomalies or aneuploidies; pregnant women who delivered at the Dr. Balmis General University Hospital but whose pregnancy was monitored in another health department; pregnant women who did not undergo first-trimester PE screening; and pregnant women with language barriers who did not have access to an interpreter.

Pregnant women with autoimmune, oncological, neurodegenerative, COVID-19, and other severe chronic diseases were not excluded from the study.

### 2.3. Study Variables

The study parameters included maternal and neonatal clinical variables and biophysical and biochemical parameters, as well as obstetric and perinatal outcomes. The primary outcome variable was the development of late-onset PE. Maternal characteristics were recorded, including demographic and epidemiological data such as age, ethnicity (Caucasian, Afro-Caribbean, Asian, Moroccan), height (cm), weight (kg), body mass index (BMI) (kg/m^2^), toxic habits (tobacco, alcohol, and/or drugs), parity (number of previous births), and family history of PE. In addition, maternal comorbidities were documented, including chronic hypertension, a previous history of PE and/or fetal growth restriction (FGR), pregestational diabetes, antiphospholipid syndrome, renal disease, and systemic lupus erythematosus.

Clinical data related to pregnancy were also collected, including the mode of conception (spontaneous or pregnancy following assisted reproductive technology), maternal weight gain during pregnancy (kg), aspirin (ASA) intake, and gestational age at delivery (weeks and days).

The biophysical parameters included the systolic blood pressure (SBP), diastolic blood pressure (DBP), and MAP measured in the first (11 + 0–13 + 6), second (19 + 0–21 + 6), and third trimesters (34 + 0–36 + 6), as well as the mean UtA-PI measured through ultrasounds in each trimester.

The biochemical parameters comprised PlGF (pg/mL), assessed at 8 + 6–13 + 6 and 34 + 0–36 + 6 weeks of gestation; sFlt-1 (pg/mL) and the sFlt-1/PlGF ratio, determined at 34 + 0–36 + 6 weeks; and serum creatinine (mg/dL) and proteinuria, obtained during laboratory testing in the third trimester.

Obstetric outcomes were recorded, including pregnancy-related complications such as gestational diabetes, gestational hypertension, oligohydramnios, intrahepatic cholestasis of pregnancy, and/or FGR (defined as an estimated fetal weight below the 3rd percentile or below the 10th percentile, in combination with abnormal fetoplacental Doppler findings [20]), as well as maternal admission to the intensive care unit.

The perinatal outcomes included neonatal sex, birth weights (g), birth weight percentiles, small-for-gestational-age neonates (defined as a birth weight < 10th percentile), admissions to the neonatal intensive care unit, and intrauterine fetal death.

### 2.4. Procedure

The women were recruited at their routine visits to the obstetrician in the third trimester (34 + 0–36 + 6 weeks of gestation). Prior to the third-trimester ultrasound examination, this study was explained both orally and in writing to eligible women, and those who agreed to participate provided their written informed consent, in accordance with the approval of the Research Ethics Committee for Medicinal Products of the hospital (CSV:I7N9CX3M:D1SNRYZA:L1VUGX8U). During the ultrasounds, the UtA-PI was specifically measured for this study via a transabdominal approach using a Voluson S8 ultrasound system (GE HealthCare, Chicago, IL, USA) equipped with multifrequency transabdominal and transvaginal transducers, with color and pulsed Doppler. Mean UtA-PI was derived from bilateral uterine artery PI measurements as (right + left)/2. Examinations were performed by four obstetricians with extensive expertise in obstetric ultrasounds, all certified with Level III Accreditation in Ultrasound by the Spanish Society of Gynecology and Obstetrics (SEGO). At the same visit, blood pressure was recorded. Measurements were obtained automatically using a calibrated OMRON M6 (OMRON Health Care Europe, BV, Hoofddorp, The Netherlands) device under standardized conditions: taking a single measurement in one arm (right or left) while the participant was seated after 5 min of rest. The mean arterial pressure was calculated as MAP = DBP + (SBP − DBP)/3. Both the UtA-PI and blood pressure results were documented in the medical records of all participants. PlGF and sFlt-1 were determined from the blood samples routinely collected in the third trimester. For this study, angiogenic factors were quantified using the same sample obtained for standard biochemical testing; therefore, participation did not require an additional venipuncture. The PlGF and sFlt-1 concentrations were measured using a sandwich immunoassay with electrochemiluminescence detection on the Cobas 8000 e602 module (Roche Diagnostics^®^, Rotkreuz, Switzerland).

### 2.5. Risk Assessment of Late-Onset Preeclampsia

The results of screening for late PE using the FMF algorithm were obtained retrospectively at the end of pregnancy by entering the clinical, biophysical and biochemical data for each participant from 30 + 0 to 37 + 6 weeks into the freely accessible online calculator (https://fetalmedicine.org/research/assess/preeclampsia/third-trimester (accessed on 1 December 2023)). The following variables were introduced into the calculator:

- Maternal factors: date of birth (dd-mm-yyyy), ethnicity (White, Black, South Asian, East Asian or Mixed), height (cm), weight (kg), currently smoking (Yes/No), conception method (spontaneous, ovulation drugs or in vitro fertilization), family history of PE (Yes/No), and parity (Nulliparous or Parous); for multiparous women, additional data from the previous pregnancy were recorded, including history of preeclampsia (Yes/No), date of delivery (dd-mm-yyyy), and gestational age at delivery (weeks and days). Systemic conditions were also documented, including pregestational diabetes (Yes, type I or II)/No), chronic hypertension (Yes/No), personal history of PE (Yes/No), systemic lupus erythematosus (Yes/No), and antiphospholipid syndrome (Yes/No).
- Biophysical parameters: right uterine artery pulsatility index (PI), left uterine artery PI, MAP (mmHg), and date of measurement of biophysical parameters.
- Biochemical parameters: sFlt-1 (pg/mL) in the third trimester, PlGF (pg/mL) in the third trimester, and data from biochemical parameter measurements.

Multiples of the median (MoM) were calculated using the batch calculation tool provided by the FMF’s website (https://fetalmedicine.org/research/mom (accessed on 1 December 2023)). Women were classified as high-risk for developing late-onset PE using the ≥1/20 cutoff proposed by Panaitescu A [8].

### 2.6. Diagnostic Criteria

Diagnoses were confirmed through a review of the medical records of all participants. The criteria established by the International Society for the Study of Hypertension in Pregnancy (ISSHP) were applied to defining late-onset PE [21]. Late-onset PE was defined as the development of new-onset hypertension associated with at least one of the following: proteinuria; clinical or laboratory evidence of maternal organ dysfunction; and/or uteroplacental dysfunction. Hypertension was defined as an SBP ≥ 140 mmHg or a DBP ≥ 90 mmHg, documented at rest on at least two occasions separated by a minimum of 4 h and occurring after 34 weeks of gestation in previously normotensive women. Proteinuria was qualitatively defined as the presence of two or more “+” results for proteins on urine dipstick testing of a single sample, in the absence of a urinary tract infection and in the context of an elevated blood pressure. Early-onset PE was excluded, as participant recruitment was conducted during the third-trimester ultrasound, which was performed between 34 + 0 and 36 + 6 weeks of gestation. Neonatal birth weight percentiles were calculated using the gestational calculator of Vall d’Hebron Hospital (https://www.medfetal.org/calculadora-gestacional/ (accessed on 1 January 2024)) based on neonatal sex, birth weight (g), and gestational age at delivery (weeks and days). Neonates were classified as small-for-gestational-age when their birth weight was below the 10th percentile according to the customized growth charts from Vall d’Hebron [22].

### 2.7. Statistical Analysis

For the statistical analysis, descriptive statistics were calculated for all study variables. Qualitative variables were summarized as the absolute frequencies and percentages. The normality of quantitative variables was assessed using the Kolmogorov–Smirnov test. Variables with a normal distribution (*p* ≥ 0.05) were expressed as the mean and the standard deviation, whereas those with a non-normal distribution (*p* < 0.05) were expressed as the median and the interquartile range.

Factors associated with the presence of late-onset PE were analyzed using contingency tables, applying Fisher’s test to categorical variables and the Mann–Whitney U test to continuous variables.

A predictive model for late-onset PE was constructed by fitting multivariate logistic regression models. Odds ratios with 95% confidence intervals (CIs) were estimated. A stepwise variable selection procedure based on the Akaike Information Criterion (AIC) was applied. The model’s fit and predictive performance were assessed through indicators such as the receiver operating characteristic (ROC) curve. Internal validation was performed using 10-fold cross-validation with 100 repetitions, reporting the area under the ROC curve and its 95% CI.

All statistical analyses were conducted using SPSS software version 28 and R software version 4.3.1.

## 3. Results

### 3.1. The Characteristics of the Study Population

**Figure 1:**
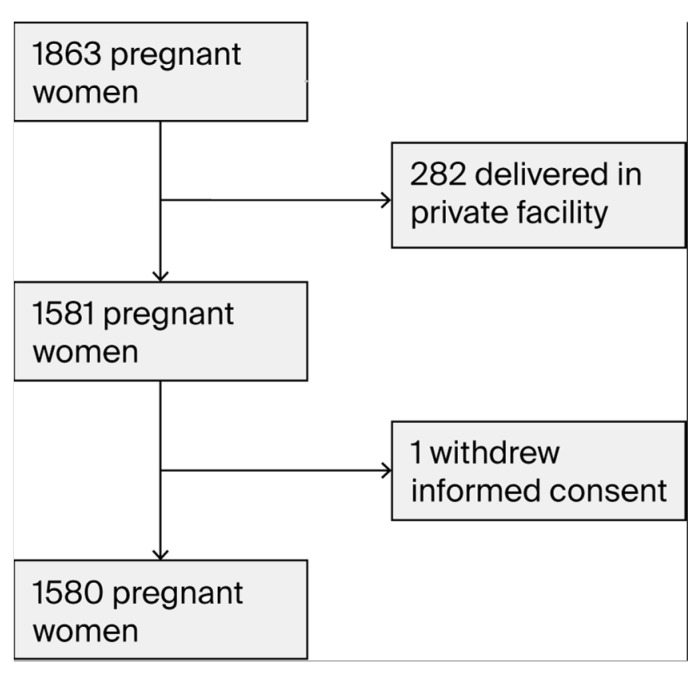
A flow chart for the inclusion/exclusion of the patients in this study.

A total of 1863 pregnant women were included. Obstetric outcomes could not be obtained for all of the participants, as delivery data for 282 women who delivered in private institutions were not available in the electronic medical record system. Ultimately, complete datasets were collected for 1581 women, 1 of whom subsequently withdrew from this study. Therefore, the final study population consisted of 1580 pregnant women (Figure 1). Of these, 46 developed late-onset PE, while 1534 women were without this disease (2.9% vs. 97.1%).

Maternal characteristics, clinical pregnancy data, biophysical and biochemical markers, obstetric outcomes, and perinatal outcomes stratified by the presence or absence of late-onset PE are presented in Table 1.

### 3.2. The Predictive Performance of the Fetal Medicine Foundation’s Third-Trimester Model for Late-Onset Preeclampsia

**Figure 2:**
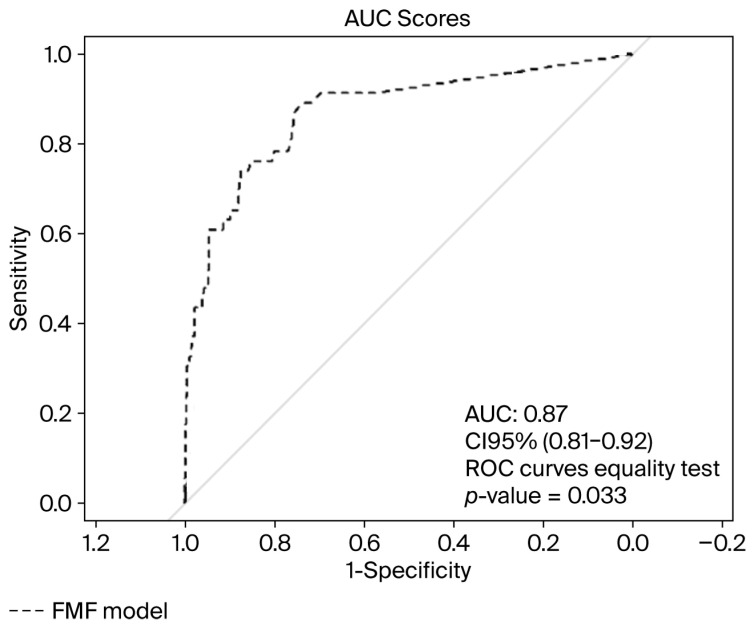
The ROC curve of the FMF model for late-onset PE prediction.

In our study population, the Fetal Medicine Foundation’s model achieved an area under the ROC curve (AUC) of 0.87, with a 95% CI = 0.81–0.92, for the classification of late-onset cases of PE. Figure 2 displays the ROC curve of the algorithm for the prediction of late-onset PE.

#### External Validation of the Fetal Medicine Foundation’s Third-Trimester Model in Our Population of Pregnant Women

For the FMF algorithm, a cutoff value of ≥1 in 20 was considered as indicative of a high risk of late-onset PE [8]. Table 2 shows the predictive performance of the model using the cutoff point recommended by the author of the algorithm.

### 3.3. Improvement of the Fetal Medicine Foundation’s Third-Trimester Model

#### 3.3.1. Modification of the Cutoffs for Our Population of Pregnant Women

Table 3 presents the predictive performance of the FMF algorithm when applying different risk cutoffs in our study population.

#### 3.3.2. The Incorporation of Additional Variables Not Included in the Original Model

We explored whether the incorporation of additional variables not included in the original FMF algorithm could improve its predictive performance. The only variable with potential added value was ASA intake. When ASA intake was introduced into the model, it demonstrated some independent explanatory capacity for late-onset PE. However, it did not enhance the overall predictive performance of the FMF algorithm.

The AUC of the crude model was 0.871 (95% CI: 0.813–0.929), whereas the multivariate model, including ASA intake, achieved an AUC of 0.872 (95% CI: 0.813–0.930), showing no significant improvement (Table 4).

### 3.4. The Development of Our Own Predictive Model

The optimal multivariate logistic model for predicting late-onset PE was developed, including variables significantly associated with the disease. Eight variables were retained: maternal age; body mass index; the presence of systemic disease (defined as pregestational diabetes, chronic hypertension, a history of PE and/or FGR, antiphospholipid syndrome, and/or chronic kidney disease); conception via in vitro fertilization; gestational weight gain; a diagnosis of gestational diabetes; the sFlt-1/PlGF ratio in the third trimester; and diastolic blood pressure in the third trimester.

The predictive performance of our predictive model for late PE, as measured using the AUC, was 0.941 (95% CI: 0.915–0.967).

Following internal validation using cross-validation with 100 repetitions and 10 folds, the honest AUC for classifying observed cases of late PE was 0.927 (95% CI: 0.840–1.000).

Once the effect of the variables has been preliminarily explored, the probability of late PE appearing is given by the following formula:

$$P(late\;PE)=\frac{1}{1+e^{-A}}$$

where

A = −20.422 + 2.030 × SD + 1.107 × GD + 1.324 × ART + 0.144 × GWG + 0.037 × RATIO + 0.169 × DBP − 0.092 + AGE + 0.111 × BMI

The variables included in the equation were

- SD: Systemic disease (pregestational diabetes, chronic hypertension, a personal history of PE and/or FGR, antiphospholipid syndrome, and/or kidney disease) (0 = no; 1 = yes);
- GD: Gestational diabetes (0 = no; 1 = yes);
- ART: Assisted reproductive technology (0 = no; 1 = yes);
- GWG: Gestational weight gain (kg);
- RATIO: The sFlt-1/PlGF ratio in the third trimester;
- DBP: Diastolic blood pressure (mmHg) in the third trimester;
- AGE: Maternal age (years);
- BMI: Body mass index (kg/m^2^).

This formula can be applied in the third trimester to calculating the risk of late-onset PE. The resulting probability (P) ranges between 0 and 1, where values close to 0 indicate a low risk and values approaching 1 indicate a high risk of developing late PE.

#### Predictive Indicators of Our Model According to Different Cutoffs

Table 5 shows the predictive indicators of the proposed model; namely, the sensitivity (Se), specificity (Sp), positive predictive value (PPV), negative predictive value (NPV), false positive rate (FPR), positive likelihood ratio (LR+), and negative likelihood ratio (LR−) for the different probability cutoffs.

The choice of the cutoff point is arbitrary, and the decision depends on the prevalence of the disease in the screened population, as well as on acceptable health and economic costs. In clinical practice, the cutoff would be defined a priori according to these indicators.

For example, using a cutoff of 1/20 (0.05)—that is, classifying a pregnant woman as high-risk for late-onset PE if the probability is greater than 0.05—the model yielded a sensitivity of 76.1%, a specificity of 91.6%, an FPR of 8.2%, an false negative rate of 23.9%, a PPV of 21.3%, an NPV of 99.2%, an LR+ of 9.0, and an LR− of 0.261. Conversely, at a cutoff of 1/10 (0.10), the sensitivity was 69.6%, the specificity was 96.0%, the FPR was 3.9%, the false negative rate of 30.4%, the PPV was 34.0%, the NPV was 99.1%, the LR+ was 17.4, and the LR− was 0.317.

The recommended cutoff point for using the proposed model in routine clinical practice would be 0.05 (1/20), as this aligns best with the false positive rate recommended for screening for this condition (around 10%).

## 4. Discussion

The prevalence of late-onset PE reported in our study is higher than that which we have observed in previous years at the Department of Health of Alicante, Dr. Balmis General University Hospital (1.7% in 2019), and higher than that reported in other series published [8,15]. This increase may have been influenced by the implementation of early PE screening in our department since 2021. According to Nicolaides, early PE screening and the administration of ASA to pregnant women at a high risk of developing this complication can “postpone” the onset of PE. Consequently, preventing early-onset PE would result in an increase in late-onset PE [23]. Another relevant factor that may have influenced this finding is the specific characteristics of the population included in this study. These results highlight the importance of considering the particularities of each context when interpreting variations in the prevalence of late-onset PE.

### 4.1. A Comparative Analysis According to the Development of Late Preeclampsia and Its Absence

Although no statistically significant differences in maternal age were observed between women who developed late-onset PE and those who did not (31.78 ± 6.52 vs. 32.01 ± 5.83), this finding is consistent with previous studies [8,15].

The study groups (i.e., those without late-onset PE vs. those with late-onset PE) did not differ with regard to the level of statistical significance (*p* > 0.05) in race/ethnicity, family history of PE, chronic hypertension, or smoking habits. These findings differ from those reported in the literature. In our study, this lack of significant differences is likely attributable to the limited number of women with variation in these characteristics. A larger sample size might have revealed significant differences, consistent with those found by other authors [8,15,17,24], given that being black, having a family history of PE, and having chronic hypertension are well-documented risk factors for the development of late-onset and term PE. Conversely, some authors have suggested that smoking may act as a protective factor against the development of this disease [8,15].

Nevertheless, a higher risk of developing late-onset PE was observed in nulliparous women; those with a high BMI; those with a personal history of PE and/or FGR; those with systemic disease; women who had taken ASA during pregnancy; and women whose pregnancies were achieved using assisted reproductive technologies. These results are consistent with those from the published literature, since all of these variables are well-established risk factors for late-onset PE [8,14,15,25,26].

Although no statistically significant differences in weight gain were observed in our study between women who developed late-onset PE and those who did not, Robillard et al. [27] and Hung et al. [28] reported significantly greater weight gain in women who developed late-onset PE. Confounding variables such as pre-pregnancy BMI may have influenced our results, emphasizing the importance of properly adjusting for confounders in future studies.

Women who developed late-onset PE presented with higher values in their biophysical markers (UtA-PI, MAP, SBP, and DBP) in all three trimesters compared with those in women who did not develop this disease. This finding was consistent with that in previous studies [8,13,14,29,30].

Overall, the biochemical marker profile of the women with late-onset PE showed significantly higher mean levels of sFlt-1 and higher sFlt-1/PlGF ratios alongside significantly lower mean PlGF levels in both the first and third trimesters of pregnancy, in line with published evidence [8,14,29,31].

Regarding the obstetric outcomes, the incidence of late-onset PE was significantly higher in women with gestational diabetes. These results are consistent with the published literature, as gestational diabetes is known to increase the risk of developing this disease [27,28]. The proportion of fetuses with FGR was also significantly higher in the group with late-onset PE, which is consistent with the clear association between FGR and both early- and late-onset PE [32].

With respect to the perinatal outcomes, the weight and birthweight percentiles of the newborns in the late-onset PE group were significantly lower. Likewise, the proportion of small-for-gestational-age newborns was significantly higher in the women with late-onset PE. Our findings are consistent with those of most previous studies [17,28], which have demonstrated that women with late-onset PE are at a greater risk of delivering small-for-gestational-age newborns compared with this risk in women without PE.

### 4.2. The Predictive Performance of the Fetal Medicine Foundation’s Third-Trimester Model for Late-Onset Preeclampsia

In our study, according to the AUC values obtained (0.87), the FMF algorithm performed well for the prediction of late-onset PE in our population of pregnant women. We attribute this good predictive capacity to the fact that the model is applied in the third trimester of pregnancy and incorporates angiogenic markers, optimizing the prediction of late-onset PE [8,17,33]. Previous studies, such as that by Panaitescu et al. [8], support these results, demonstrating that combined screening performed between 35 and 37 weeks of gestation achieves a superior performance compared to that of screenings conducted at 11–13, 19–24, and 30–34 weeks, with significantly higher detection rates. For example, performing a combined screening between 30 and 34 weeks achieves a detection rate (DR) of approximately 65%, while screenings performed in the first or second trimester reach detection rates of 45% [34,35,36].

#### External Validation of the Fetal Medicine Foundation’s Third-Trimester Model in Our Population of Pregnant Women

Validating models in geographically diverse populations is an appropriate strategy for assessing their reproducibility [37,38]. For this reason, we decided to validate the performance of the FMF algorithm [8] in our population of pregnant women.

The FMF model for the third trimester of pregnancy was previously validated by Döbert et al. [15]; however, although Spanish pregnant women were included in their validation study, how the algorithm specifically performed in this population was unclear, as the number of Spanish women included was not reported. In this regard, our study provides a relevant contribution by analyzing the performance of the algorithm using the cutoff proposed by the author [8] (1/20) in a predominantly Spanish population of pregnant women, which is of interest for clinical practice at the national level.

Despite the low sensitivity of 32.6% obtained using the recommended cutoff [8], which reflects the limited capacity of the algorithm to detect pregnant women who will develop late-onset PE in our setting, the specificity was very high, at 98%, making it appropriate for correctly identifying women who will not develop late-onset PE. Meanwhile, the FPR in our study was low, at 1.3%.

Nevertheless, the PPV was poor, meaning that the algorithm was not able to reliably identify women who would develop late-onset PE as high-risk [39,40]. Conversely, the NPV was very high (98%), indicating that the algorithm is reliable for classifying women who will not develop late-onset PE as low-risk.

The LR+ obtained using the FMF algorithm indicates that a pregnant woman with late-onset PE is 23 times more likely to obtain a positive screening result than a woman who will not develop this disease. According to the reference standards [41,42], this value reflects the excellent ability of the algorithm to confirm positive cases. However, the LR− was suboptimal, which limits its utility as a screening tool to confidently rule out late-onset PE in women classified as low-risk in our population (Table 2).

Compared with the existing literature, in our study, the DR was notably lower than that reported by the authors of the FMF algorithm [8]. Using combined screening of maternal factors between 35 and 37 weeks of gestation, the MAP, the UtA-PI, PlGF, and sFlt-1 and applying a cutoff of a “risk greater than 1 in 20” to classifying cases of late-onset PE, the original authors of the FMF algorithm obtained a DR of 68.8%, with an FPR of 9.1%. In contrast, using the same cutoff [8], we obtained a considerably lower DR of 32.6% in our population, with an FPR of 1.3%.

Other studies, such as that by Andrietti et al. [14], reported a DR of 84% with an FPR of 10%, while validation studies, such as Döbert et al.’s [15] external validation of the FMF algorithm in English, Belgian, and Spanish populations, reported a DR higher than ours, reaching 79%, with an FPR of 10%.

These discrepancies highlight the lack of reproducibility of the FMF’s third-trimester algorithm in our population of pregnant women.

In conclusion, although the FMF algorithm may represent a potentially valuable tool for screening for late-onset PE, its limited sensitivity at the cutoff proposed by its authors [8] and the discrepancies in the detection rates in our population underscore the need for refinement. These findings highlight the importance of adjusting the proposed cutoffs or developing a predictive model tailored to the characteristics of our specific population.

### 4.3. Improvement of the Fetal Medicine Foundation’s Third-Trimester Model

#### 4.3.1. Modification of the Cutoffs for Our Population of Pregnant Women

The clinical utility of the FMF’s third-trimester algorithm largely depends on the established cutoffs and their suitability for the prevalence of late-onset PE in each specific population.

Given the low DR obtained using the recommended cutoff [8], we proposed alternative cutoffs of 1/100, 1/150, and 1/200 (Table 3) with the aim of increasing the sensitivity, even though this would entail a reduction in specificity and consequently an increase in the FPR. Nevertheless, since the FPR for the original cutoff was low, we were willing for it to increase slightly.

With the FMF algorithm, all of the cutoffs that we proposed were more sensitive than the original (Table 3). The optimal cutoff for applying this model in our population to routinely screen for late-onset PE in the third trimester was determined to be 1/200. Although this cutoff involves a higher FPR, it provides a substantially greater sensitivity, nearly twice that achieved using the original cutoff, thereby allowing the greatest number of pregnant women in our population who will develop late-onset PE to be identified. Furthermore, the FPR of 8.3% obtained is still slightly lower than the rate generally considered acceptable for screening for this condition, at approximately 10% [8,14,15,30].

#### 4.3.2. The Incorporation of Additional Variables Not Included in the Original Model

According to Nicolaides et al. [23], ASA intake may “delay” the onset of PE, preventing early-onset PE but not late-onset PE. For this reason, we decided to assess whether incorporating ASA intake as a variable could improve the predictive capacity of the FMF model [8].

It was observed that although ASA intake was included in the model and demonstrated independent explanatory value for late-onset PE, it did not significantly improve the predictive capacity of the FMF model. This finding is evidenced by the values of the area under the ROC curve, which remained virtually unchanged between the crude model (0.871) and the multivariable adjusted model that included ASA intake (0.872). The absence of an improvement in the predictive performance may be explained by the high complexity and effectiveness of the original model, which integrates maternal factors, biophysical parameters (MAP and UtA-PI), and biochemical markers (sFlt-1 and PlGF) for the prediction of late-onset PE, a combination of predictors with substantial explanatory power.

### 4.4. The Development of Our Own Predictive Model

The prediction of late-onset PE represents a major challenge, particularly in populations where the existing predictive models, such as that developed by the FMF, have not achieved an adequate performance when applying the cutoff originally proposed by the author [8]. This limitation highlights the need to develop screening tools tailored to the specific characteristics of each population.

In this context, we developed our own multivariable model designed for implementation in the third trimester of pregnancy, with the objective of improving predictive performance for late-onset PE in our population of pregnant women. This model integrated important predictors of late-onset PE previously identified in the literature. From a set of preselected variables, eight were found to be statistically significant and were assigned relative weights, as defined in the model equation.

The variables included were maternal age, BMI, the presence of systemic disease (pre-gestational diabetes, chronic hypertension, personal history of preeclampsia and/or FGR, antiphospholipid syndrome and/or renal disease), a diagnosis of gestational diabetes during pregnancy, conception through assisted reproductive technologies, gestational weight gain, diastolic blood pressure in the third trimester, and the sFlt-1/PlGF ratio in the third trimester.

Our findings aligned with those of previous studies, as all of the variables we included have been reported as risk factors for late-onset PE [8,14,15,17,28]. Furthermore, in the bivariate analysis (Table 1), most of these variables showed significant differences between the group of women who developed late-onset PE and those who did not, except for gestational weight gain and maternal age.

It is noteworthy that although gestational weight gain was not significant in the bivariate analysis (Table 1), it became significant in the multivariable analysis. This finding reflects confounding bias with other variables. In the bivariate analysis, such bias may conceal the true associations between weight gain and late-onset PE, whereas in the multivariable analysis, adjusting for confounding variables eliminates this bias and reveals the significant association.

This finding underscores the importance of interpreting the results of a bivariate analysis with caution, as they may be influenced by such biases. In observational studies such as ours, a multivariable analysis is essential to obtaining a more precise and adjusted estimation of the relationships among the variables studied.

Additionally, our model identified relevant parameters as predictors of late-onset PE, such as gestational diabetes and gestational weight gain, which had not been previously included in predictive models for the third trimester [8,14,33]. These variables add further value to the existing predictive approach.

Our model also reinforces the importance of implementing combined screening in the third trimester that integrates angiogenic markers such as PlGF and sFlt-1 as predictive variables, thereby enhancing the predictive performance for late-onset PE. Our findings are aligned with those for algorithms such as that from the FMF which already incorporate these factors into the combined screening [8,15,33].

Some variables, such as the mean uterine artery pulsatility index measured in the third trimester, which showed a significant association with late-onset PE in the bivariate analysis (Table 1) were not retained in our model. This indicates that the variable did not have an independent explanatory capacity; that is, other variables in the model overlapped with its explanatory role, meaning they accounted for the same predictive contribution as that of the UtA-PI.

This finding is consistent with the literature. For example, O’Gorman et al. [43] included this parameter in their model for assessing the risk of preterm PE; however, it did not improve their model when it was applied to predicting term PE. Similarly, studies by Panaitescu et al. [8] and Döbert et al. [15] support our results, emphasizing that the best predictive performance is achieved by combining maternal factors, MAP, PlGF, and sFlt-1, with no additional benefit of including the UtA-PI for the prediction of late-onset PE.

Excluding the mean UtA-PI from our screening algorithm may offer a practical advantage by simplifying the model implementation and reducing the time required for patient evaluations. In addition, the quality and accuracy of UtA-PI measurements often depend on the experience and skill of the obstetrician, which may lead to variability in the results.

In terms of performance, our predictive model achieved an area under the ROC curve of 0.941, with a 95% CI = 0.915–0.967, reflecting an excellent predictive capacity [44]. As expected, the AUC decreased to 0.927 when validated internally using cross-validation techniques. This AUC is close to that expected if the model were applied to an external population of pregnant women. This result is also comparable to that reported by Döbert et al. [15], who found a similar AUC of 0.938 in their study internally validating the FMF algorithm.

When compared with that of the FMF algorithm, the AUC obtained for our predictive model was significantly higher (0.94 vs. 0.87). Therefore, our results demonstrate a marked improvement in the predictive performance relative to that of the preexisting algorithm [8].

The main limitation of our model lies in the relatively small number of late-onset PE cases in our dataset. According to the literature, model adjustment to the study population should ideally include 1 variable per 10 events [45]. In our case, the model was slightly overfitted, with 8 variables for 46 cases of late-onset PE, instead of the 4 or 5 variables recommended by the general guidelines. This proportion suggests that our model may be slightly overestimated.

Nevertheless, it is important to highlight that our model is robust, as the variables included are statistically significant and demonstrate a strong predictive ability for the development of this disease.

Although the internally developed and validated model demonstrated a good performance, external validation in a different population remains necessary.

Despite its strengths, including its high predictive capacity and simplified structure facilitating clinical implementation, it is essential to conduct prospective studies using larger sample sizes to externally validate our model and confirm its performance in other populations of pregnant women.

Another consideration that may hinder its implementation in routine clinical practice is the high cost associated with the determination of biochemical markers, particularly in health systems with budgetary limitations or low-resource settings. Therefore, cost effectiveness studies are warranted to support or justify its adoption in routine care.

In conclusion, this predictive model represents a promising tool for screening for late-onset PE. Considering the above, it may be consolidated into an effective algorithm for improving the management of pregnant women at risk of late-onset PE, thereby optimizing maternal and perinatal outcomes.

#### Predictive Indicators of Our Model According to Different Cutoffs

Table 5 facilitates an evaluation of our own model from another perspective, perhaps a more practical perspective from a clinical point of view, as it presents the performance of our predictive model for the different proposed cutoff points.

The recommended cutoff for the application of our model in routine clinical practice in our population of pregnant women would be 1/20 since this offers the highest sensitivity, minimizing false negatives and ensuring that most women who will develop late-onset PE are identified in advance. These women could thus benefit from closer clinical surveillance and programmed delivery, reducing both maternal and fetal complications. Moreover, it would allow women at an increased lifetime risk of cardiovascular complications to be identified, in whom preventive measures should be initiated preemptively.

Although this cutoff involves a reduction in specificity and a slight increase in the FPR (8.2%), the latter value is deemed acceptable as it remains below the 10% threshold generally recommended by authors for screening for this condition [8,14,15,30]. This ensures that the negative impact associated with misclassification is minimal and manageable in clinical practice.

### 4.5. Strengths and Limitations

One of the main strengths of this study is the prospective inclusion of pregnant women.

In addition, this study was conducted within the framework of routine clinical practice and women were attended by their usual healthcare providers, increasing the reliability and applicability of the results in real-world healthcare settings.

Another strength of this work is that it constitutes the first study to evaluate the performance of the FMF’s third-trimester algorithm exclusively in a cohort of Spanish pregnant women.

Beyond the considerations regarding our own predictive model, one of the main limitations is that this was a single-center study, including only women from our Department of Health. This characteristic limits the generalizability of the findings, as the results may not be fully extrapolatable to pregnant women with markedly different sociodemographic characteristics from those of our cohort. Therefore, although our model demonstrated a good performance in our population, its applicability to other contexts requires external validation.

## 5. Conclusions

For the FMF algorithm, we recommend a cutoff of 1/200 for the routine screening of late-onset PE in our population, as this achieves a better balance between the DR and the FPR.

Our screening model demonstrated a higher predictive capacity than that of the other model we evaluated. It therefore constitutes a promising tool for screening for late-onset PE during the third trimester in our population. Its high predictive performance and the simplified approach of excluding the mean pulsatility index in the uterine arteries facilitates its implementation in routine clinical practice.

## Figures and Tables

**Table 1:** The characteristics of the study population.

	No Late PE (n= 1534)	Late PE (n= 46)	*p*-Value
**Maternal characteristics**			
**Demographic and epidemiological data**			
Maternal age in years	32.01 ± 5.83	31.78 ± 6.52	0.838
Racial origin			
Caucasian	1497 (97.59)	46 (100)	0.624
Other	37 (2.41)	0 (0.0)	
Maternal body mass index, kg/m^2^	25.20 ± 4.87	28.35 ± 6.75	**0.002**
Smoking	171 (11.15)	3 (6.52)	0.472
Family history of PE	40 (2.61)	3 (6.52)	0.127
Parity (number of previous deliveries)			
None	790 (51.50)	33 (71.74)	**0.032**
One	521 (33.96)	12 (26.09)	
Two	163 (10.63)	1 (2.17)	
Three or more	60 (3.91)	0 (0)	
**Maternal comorbidities**			
Chronic hypertension	29 (1.89)	2 (4.34)	0.227
Previous PE and/or FGR	39 (2.54)	6 (13.04)	**0.002**
Systemic disease	77 (5.02)	9 (19.57)	**0.001**
**Clinical pregnancy data**			
Assisted reproduction			
NO	1447 (94.33)	39 (84.78)	**0.017**
SI	87 (5.67)	7 (15.22)	
Gestational weight gain (kg)	11.31 ± 3.87	11.97 ± 6.45	0.843
Aspirin intake	39 (2.54)	9 (19.57)	**<0.001**
**Biophysical markers**			
SBP (mmHg)			
1st T	117.51 ± 11.50	123.61 ± 10.89	**0.001**
2nd T	112.52 ± 9.47	124.89 ± 9.00	**<0.001**
3rd T	116.65 ± 11.01	132.87 ± 14.76	**<0.001**
DBP (mmHg)			
1st T	73.93 ± 8.70	78.52 ± 8.89	**0.001**
2nd T	71.84 ± 8.48	77.63 ± 6.42	**<0.001**
3rd T	75.60 ± 7.94	87.30 ± 8.37	**<0.001**
MAP (mmHg)			
1st T	88.46 ± 8.52	93.55 ± 8.27	**<0.001**
2nd T	85.40 ± 7.74	93.38 ± 6.46	**<0.001**
3rd T	89.28 ± 7.93	102.49 ± 9.46	**<0.001**
Uterine artery PI median			
1st T	1.38 ± 0.49	1.47 ± 0.52	0.284
2nd T	0.93 ± 0.23	1.08 ± 0.38	**0.004**
3rd T	0.69 ± 0.18	0.80 ± 0.28	**0.011**
**Biochemical markers**			
PlGF (pg/mL) 1st T	33.39 ± 16.01	25.54 ± 10.40	**<0.001**
sFlt-1 (pg/mL) 3rd T	1333.68 ± 1444.25	3630.76 ± 5379.09	**<0.001**
PlGF (pg/mL) 3rd T	437.54 ± 344.07	156.05 ± 95.06	**<0.001**
sFlt-1/PlGF 3rd T	9.54 ± 13.69	53.70 ± 64.55	**<0.001**
**Obstetric outcomes**			
Gestational diabetes	152 (9.91)	14 (30.43)	**<0.001**
FGR	33 (2.15)	5 (10.87)	**0.004**
**Perinatal outcomes**			
Birth weight (g)	3304.40 ± 420.58	2973.15 ± 575.75	**<0.001**
Low birth weight	320 (20.86)	21 (45.65)	**<0.001**
Birth weight percentile	34.52 ± 26.31	28.15 ± 29.27	**0.015**

Values are expressed as the mean ± standard deviation or the number (percentage). Comparisons between late-onset PE and categorical variables were performed using Fisher’s exact test, and continuous variables were compared using the Mann–Whitney U test. Statistically significant *p*-values (*p* < 0.05) are highlighted in bold. The variable “systemic disease” included chronic hypertension, a history of preeclampsia and/or fetal growth restriction, pregestational diabetes, antiphospholipid syndrome, and renal disease. PE: preeclampsia; FGR: fetal growth restriction; T: trimester; SBP: systolic blood pressure; DBP: diastolic blood pressure; MAP: mean arterial pressure; PI: pulsatility index; PlGF: placental growth factor; sFlt-1: soluble fms-like tyrosine kinase-1.

**Table 2:** The predictive performance of the FMF algorithm using the ≥1 in 20 risk cutoff.

	Estimate and 95% CI
Sensitivity (%)	32.6 (19.1–46.1)
Specificity (%)	98.6 (98.0–99.2)
PPV (%)	41.7 (25.6–57.8)
NPV (%)	98.0 (97.3–98.7)
FPR (%)	1.3
Positive LR	23.286 (12.855–42.181)
Negative LR	0.684 (0.559–0.836)

95% CI: 95% confidence interval; PPV: positive predictive value; NPV: negative predictive value; FPR: false positive rate; LR: likelihood ratio.

**Table 3:** The predictive performance of the FMF algorithm at different risk cutoffs (≥1/100, ≥1/150, and ≥1/200).

Cutoff	≥1/100	≥1/150	≥1/200
Sensitivity (%)	60.9 (46.8–75.0)	60.9 (46.8–75.0)	63.0 (49.0–77.0)
Specificity (%)	94.6 (93.5–95.7)	92.8 (91.5–94.1)	91.4 (90.0–92.8)
PPV (%)	25.2 (17.1–33.3)	20.1 (13.4–26.8)	18.0 (12.1–23.9)
NPV (%)	98.8 (98.2–99.4)	98.8 (98.2–99.4)	98.8 (98.2–99.4)
FPR (%)	5.3	7.0	8.3
Positive LR	11.278 (8.254–15.410)	8.458 (6.311–11.336)	3.326 (5.565–9.645)
Negative LR	0.413 (0.288–0.592)	0.421 (0.293–0.604)	0.405 (0.278–0.591)

The sensitivity, specificity, PPV, NPV, and FPR are expressed as percentages with 95% confidence intervals (CIs). Likelihood ratios are expressed as point estimates with 95% CIs. PPV: positive predictive value; NPV: negative predictive value; FPR: false positive rate; LR: likelihood ratio.

**Table 4:** Improvement of the Fetal Medicine Foundation model.

	Crude Adjustment	Multivariate Adjustment with ASA
AUC	0.871 (0.813–0.929)	0.872 (0.813–0.930)

AUC: area under the curve; ASA: aspirin.

**Table 5:** The predictive indicators of the model according to probability cutoffs.

Cutoff	Se (%)	Sp (%)	FPR (%)	PPV (%)	PNV (%)	LR+	LR−
0.05	76.1	91.6	8.2	21.3	99.2	9.0	0.261
0.10	69.6	96.0	3.9	34.0	99.1	17.4	0.317
0.15	54.3	97.5	2.5	39.1	98.6	21.7	0.469
0.20	45.7	98.4	1.5	46.7	98.4	28.5	0.552
0.25	45.7	98.7	1.3	51.2	98.4	35.1	0.550
0.30	43.5	99.2	0.8	60.6	98.3	54.3	0.570
0.35	41.3	99.5	0.5	70.4	98.3	82.6	0.590
0.40	39.1	99.5	0.4	72.0	98.2	78.2	0.612
0.45	39.1	99.6	0.4	75.0	98.2	97.7	0.611
0.50	37.0	99.7	0.3	81.0	98.1	123	0.632
0.55	34.8	99.9	0.1	88.9	98.1	348	0.653
0.60	30.4	99.9	0.1	87.5	98.0	304	0.697
0.65	26.1	99.9	0.1	92.3	97.8	261	0.740
0.70	23.9	99.9	0.1	91.7	97.8	239	0.762
0.75	21.7	99.9	0.1	90.9	97.7	217	0.784
0.80	17.4	99.9	0.1	88.9	97.6	174	0.827
0.85	13.0	99.9	0.1	85.7	97.5	130	0.871
0.90	13.0	99.9	0.1	85.7	97.5	130	0.871
0.95	10.9	99.9	0.1	83.3	97.4	109	0.892

Se: sensitivity; Sp: specificity; FPR: false positive rate; PPV: positive predictive value; NPV: negative predictive value; LR+: positive likelihood ratio; LR−: negative likelihood ratio.

## Data Availability

While the data has not been made publicly available to maintain patient privacy, it can be obtained by contacting the authors.

## Abbreviations

The following abbreviations are used in this manuscript:

AIC	Akaike Information Criterion
ASA	aspirin
AUC	area under the curve
BMI	body mass index
CI	confidence interval
CIs	confidence intervals
DBP	diastolic blood pressure
DR	detection rate
FGR	fetal growth restriction
FMF	Fetal Medicine Foundation
FPR	false positive rate
ISSHP	International Society for the Study of Hypertension in Pregnancy
LR−	negative likelihood ratio
LR+	positive likelihood ratio
MAP	mean arterial pressure
MoM	multiples of the median
NPV	negative predictive value
PE	preeclampsia
PI	pulsatility index
PlGF	placental growth factor
PPV	positive predictive value
ROC	receiver operating characteristic
SBP	systolic blood pressure
Se	sensitivity
SEGO	Spanish Society of Gynecology and Obstetrics
sFlt-1	soluble fms-like tyrosine kinase-1
Sp	specificity
UtA-PI	uterine artery pulsatility index
